# Renewable Polysaccharides Micro/Nanostructures for Food and Cosmetic Applications

**DOI:** 10.3390/molecules25214886

**Published:** 2020-10-22

**Authors:** Alessio Massironi, Andrea Morelli, Dario Puppi, Federica Chiellini

**Affiliations:** Department of Chemistry and Industrial Chemistry, University of Pisa, UdR INSTM-Pisa, Via G. Moruzzi 13, 56124 Pisa, Italy; alessio.massironi@dcci.unipi.it (A.M.); a.morelli@dcci.unipi.it (A.M.); dario.puppi@unipi.it (D.P.)

**Keywords:** polysaccharides, nanotechnologies, food science, cosmetics, renewable materials

## Abstract

The worldwide diffusion of nanotechnologies into products nowadays has completely revolutionized human life, providing novel comfort and benefits. Their inclusion in food and cosmetic has a heavy impact over the market, allowing the development of higher value products with enhanced properties. Natural origin polymers and in particular polysaccharides represent a versatile platform of materials for the development of micro/nanostructured additives for food and cosmetic products due to their chemical versatility, biocompatibility, and abundance. Here, we review the current applications of polysaccharides-based micro/nanostructures, taking into consideration the precursors’ production, isolation, and extraction methods and highlighting the advantages, possible drawbacks, and market diffusion.

## 1. Introduction

Additives and preservatives are commonly required to maintain the integrity of a commercial product against spoiling, extending its shelf-life for a sufficient period to accomplish the desired application [1,2,3]. In particular, additives play a fundamental role in aqueous cosmetic and food products, which are a common target of microorganisms and can present a strong oxidant environment [4]. Such additives must guarantee high resistance against degradation without modifying the taste, odor, and aspect of a product, thus maintaining all initial features. Nanotechnology is used to evolve conventional food and cosmetic additives for product preservation [5]. Micro/nanomaterials can be designed and optimized in order to offer novel value and features to an everyday product. The encapsulation of active agents in food and cosmetic products is an efficient strategy for molecular protection against chemical modification, such as oxidation, which usually causes degradation [6]. Moreover, functional nanomaterials may find application as nanosensors and tracers for the molecular recognition of possible threats contained in the final products, such as toxins or microorganisms, the presence of which could represent a possible health risk for the consumers [7]. Micro/nanoencapsulation techniques are particularly useful with oily compounds, since most natural fragrances and flavors used in the food and cosmetic industries are lipophilic molecules, which commonly display poor aqueous solubility and stability; in fact, when added to an aqueous product such as a soft drink formulation, an instantaneous phase separation occurs. A well-established procedure to enhance oily phase conservation consists of their encapsulation in micro/nanostructures through emulsification methods, which allows protection and potential exploitation, even in an aqueous environment [8].

To date, several strategies and materials have been exploited for the design and preparation of micro/nanostructures with potential applications in the food and cosmetic industries. Among them, inorganic components represent one of the most investigated materials to develop micro/nanostructures with suitable mechanical and thermal stability. Depending on the chemical nature, structure, size, and crystallinity, they can offer enhanced magnetic, electronic and/or redox properties in comparison to organic compounds [9]. Titanium and zinc oxides (TiO_2_, ZnO), silica, iron oxide (Fe_2_O_3_) and noble-metals represent the most explored materials for the development of nanostructures with potential applications in food and cosmetics. ZnO and TiO_2_ nanostructures are produced worldwide, with up to 10,000 tons of TiO_2_ nanoparticles annually synthesized, which makes them ones of the most produced metal nanomaterials [10]. Both TiO_2_ and ZnO nanostructures were mainly investigated as photocatalyst [11] and due to their strong UV absorption and reflective properties have been employed as additive components for personal care products, such as in sunscreen [12]. ZnO and TiO_2_ nanostructures have already been synthesized through several methodologies, but to date their exploitation as active agents in cosmetic formulations is hampered by high ecotoxicity [13] and possible cytotoxicity [14,15]. Noble metal nanostructures, in particular silver nanoparticles (AgNPs), exhibit a strong antimicrobial activity that has been employed for the development of advanced food packaging to avoid microbe contaminations [16,17]. The inherent optical property of gold nanoparticles (AuNPs) has been exploited to develop colorimetric sensors for food contaminant detection with potential application in rapid food safety screening [18]. Despite their well-established antimicrobial activity, the use of noble metal nanostructures in food packaging is still under debate by the scientific community since they may migrate from the packaging and containers into the foods and as a consequence be ingested by the consumers [19]. In this respect, recent studies have shown that AgNPs display toxic effects in humans when ingested at sufficiently high levels [20]. A range of marketed cosmeceutical products (e.g., deodorants and antiaging creams) take benefit from the antibacterial and antifungal properties of AuNPs as well as from other inherent properties of nanogold for beauty care, such as acceleration of blood circulation and anti-inflammatory properties [21]. However, safety concerns related to deep penetration of nanosized inorganic materials into the skin are often raised [22]. A further class of inorganic nanomaterials with potential applications in food and cosmetic industries is iron oxide nanoparticles (Fe_2_O_3_NPs), commonly exploited as a food colorant [23]. Recent studies have shown the potential application of Fe_2_O_3_ as a source of bioavailable iron in the human diet [24]; however, since iron-based nanomaterials present inherent cytotoxicity and potential side-effects to the consumer, their application in the food industry is still hindered [25]. A promising class of inorganic nanomaterials is nanodiamonds (NDs), whose biocompatibility and nontoxicity combined with fascinating physical features make them an attractive candidate for biomedical and pharmaceutical applications [26]. NDs and relevant nanomaterials show good light absorption properties, and allow enhancement of the mechanical properties of loaded compounds, as well as protection against UV irradiation and oxidation [27]. Indeed, NDs are currently investigated as materials for the development of advanced healthcare products [27]. Moreover, NDs have been exploited as novel bioactive food packaging compounds, thanks to their strong antimicrobial activity [28]. Notwithstanding their activity, synthetic micro-nanomaterials and in particular inorganic metal and carbon micro/nanomaterials are commonly developed by employing potential toxic precursors, which present high production costs; their presence could also result in intolerance, allergies, and possible side-effects to the consumer [29,30,31]. All these drawbacks can strongly affect the relevant market. Moreover, the recent growing demand by consumers for the development of greener products based on the use of natural ingredients represents a fundamental turning point toward the exploitation of new natural preservatives and additives to avoid the use of additives of synthetic origin [5,32,33]. A wide variety of biomolecules derived from plant and animal sources have been already investigated for the development of edible and biodegradable micro/nanostructures with the goal to substitute petrochemical-based additives. Among them, proteins and polysaccharides are the most exploited additive components for food and cosmetic applications, thanks to their abundance and chemical versatility [34]. Between the two natural polymers, proteins commonly present high production and conservation costs due to the low stability that limits their use. Nowadays, only collagens, silk, and milk proteins display a sustainable and advantageous procedure for the production of micro/nanostructured formulations for the food and cosmetic industries [35,36,37]. Thanks to the development of innovative, efficient, and eco-friendly extraction techniques, the isolation and purification of polysaccharides has become inexpensive, leading to straightforward and economical advantageous industrial applications.

**Figure 1:**
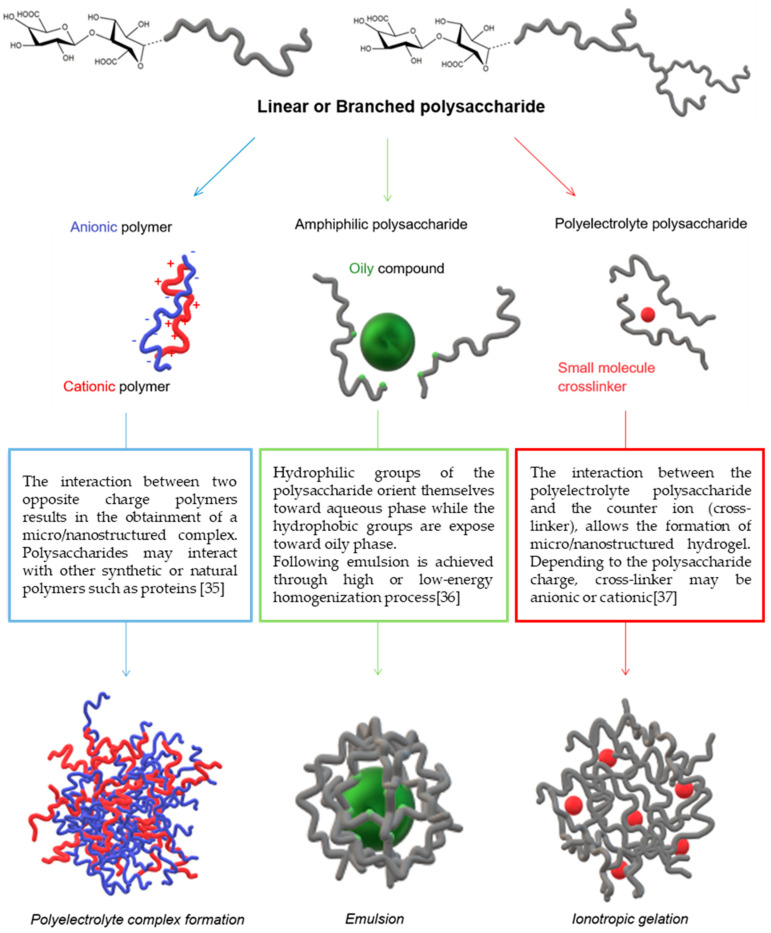
Schematic representation of polysaccharide-based micro/nanostructures’ formation.

Polysaccharide-based micro/nanostructures for food and cosmetic applications are usually achieved by following three main methodologies (Figure 1), which exploit the chemical nature of polysaccharides by interaction with:

1. Opposite charge macromolecules (polyelectrolyte complex formation) [38]
2. Lipophilic molecules (emulsification) [39]
3. Through the addition of crosslinkers (ionotropic gelation) [40]

**Figure 2:**
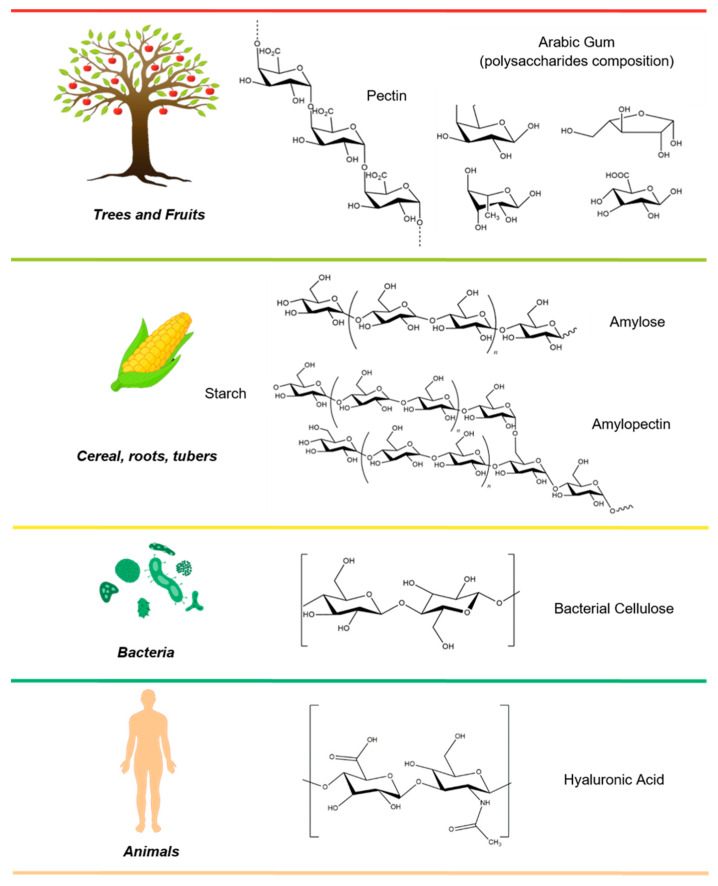
Natural source and chemical structure of polysaccharides.

**Figure 3:**
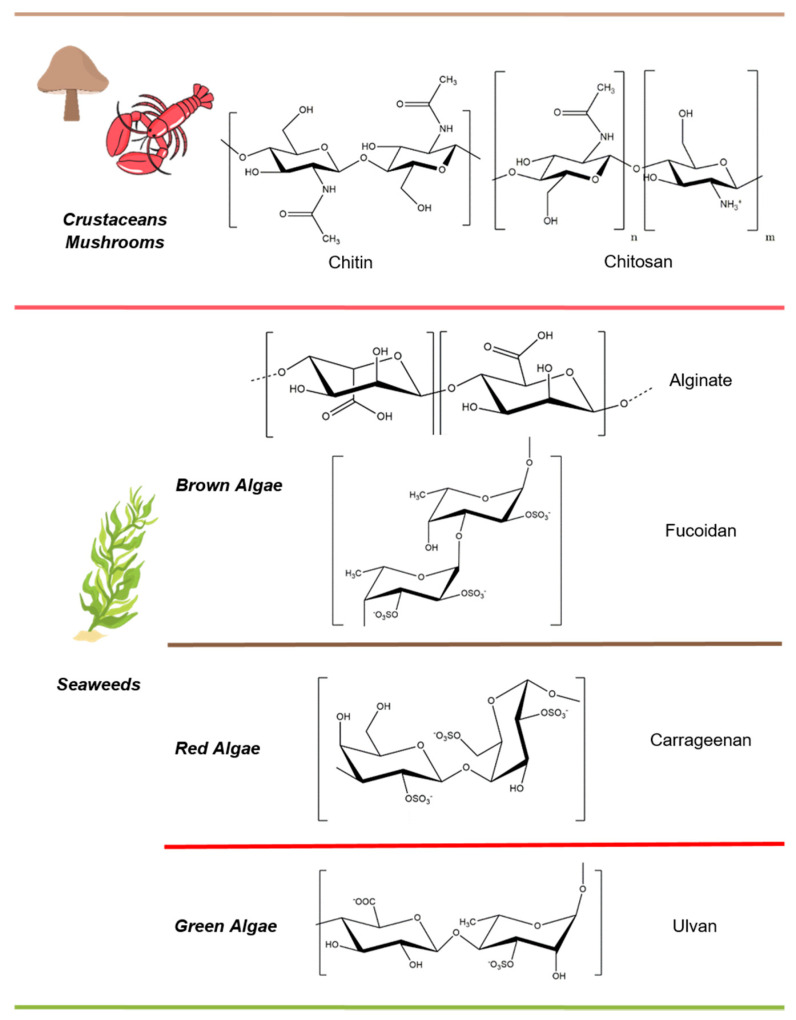
Natural source and chemical structure of marine polysaccharides.

In the present review, an overview of the most exploited polysaccharides, their chemical and/or physical modification, and processes employed for the fabrication of micro/nanostructured devices for food and cosmetic applications is presented and discussed. A schematic representation of their structures and natural sources is provided in Figure 2 and Figure 3.

## 2. Most Investigated/Exploited Polysaccharide

### 2.1. Pectin

A worldwide-diffuse and exploited polysaccharide in food industries is pectin, a heteropolysaccharide mainly composed of α-d-galacturonic acid, d-galactose, and l-arabinose residues (Figure 2). The Food and Agriculture Organization and World Health Organization FAO/WHO committee defined polysaccharides as a safe additive for food [41]. Pectin is one of the main component of the cell wall and middle lamella of several plants and fruits and its extraction is performed in mild condition using hot water. The major source of pectin is waste generated from citrus fruits, sugar beet, apple pomace, and sunflower processing; indeed, in lemon peel, the amount of polysaccharide can reach 30% of the total dry weight. Pectin is mainly used as a gelling agent in jam, jellies, conserves, and preserves; and is added as a dry powder or as a concentrated solution [35]. The use of polysaccharide in the food industry is not only limited as a gelling and thickening agent; recent studies report a potential application of pectin as a nanocarrier for bioactive molecules [42]. Pectin micro/nanostructures for food industries are obtained through several methodologies such as nanoemulsion in the presence of whey proteins for the encapsulation of essential oils and lipophilic flavors, nanocomplex formation in the presence of sodium caseinate forming a polyelectrolyte complex loading curcumin extract [43], and through nanohydrogel preparations in the presence of albumin to encapsulate vitamin C [44]. Recently, pectin expanded its range of possible applications due to its high efficacy in cosmetics industries, in particular, for personal care product formulations, demonstrating an optimal activity as a skin anti-aging agent and as a stabilizer for shampoos and lotions [45].

### 2.2. Arabic Gum

Arabic gum (also known as acacia gum) (E414) is an edible biopolymer obtained from mature trees of Acacia senegal and Acacia seyal growing mostly in the African region. This gum is one of the major natural-based additives exploited in food industries as a stabilizer, thickener, and emulsifier [46]. Arabic gum is a complex mixture of macromolecules composed of carbohydrates (mainly d-galactose and l-arabinose unit) (Figure 2) and proteins (arabinogalactan protein complex (AGP), arabinogalactan (AG) and glycoprotein); however, its composition is strongly dependent on its origin, harvest season, and tree age [47]. Nowadays, the addition of Arabic gums in food preparation represents a common procedure, but their first uses were reported in 2650 BC in ancient Egypt, when it was employed in the manufacturing of bandages for mummies and for food conservation [48]. Its amphiphilic nature made it suitable as an optimal emulsifying agent for the retention and protection of chemically reactive and volatile commercial food flavoring [49]. In particular, the biopolymer has been widely exploited in soft drinks’ formulation as a cloudy and weighting agent [46]. The ability to form a micro/nanostructure during the emulsification process is due to its structural rearrangement. In the presence of lipophilic molecules, hydrophilic groups of the polysaccharide are exposed to an aqueous phase, while the hydrophobic groups of the proteins orient themselves toward the oily phase. Over the past years, Arabic gum has also found novel applications as antioxidant agents, hampering possible food degradation and maintaining the quality of stored vegetables [50]. In this respect, a wide number of “gums”, such as xanthan (E415), gaur (E411), and locust bean (E410) are currently under investigation [51]. Nevertheless, despite its potential use, Arabic gum is classified as an additive compound, and thus, there is growing interest on the identification of novel edible agents as alternatives to Arabic gum formulation in order to reduce label specifications in the finished products [52].

### 2.3. Starch

Starch represents a plant’s energy store. It is mainly composed of a mixture of linear poly(1,4-α-d-glucopyranose) (amylose) and branched poly(1,4-α-d-glucopyranose) with branches of (1,6-α-d-glucopyranose) (amylopectin) (Figure 2). The amount and composition of the two polysaccharides may vary according to the botanical origins and harvested time and are commonly accumulated in macroscopical granules [53]. Starch nanostructures are obtained by means of hydrolysis using acids and/or enzymes or by physical treatments of granules through ultrasonication techniques [54]. Starch-based nanoparticles are currently used as fat or oil replacer in food industries [55]. Fat replacer is a food additive that imitates the organoleptic and physical properties of fatty acids such as triglycerides but decreases the final total amount of calories. The market for starch products is constantly growing due to polysaccharide versatility and possible production of nanostructures through easy methodologies exploiting industrials wastes [56]. Starch derivates obtained through the chemical or physical modification of native polysaccharide represent a further important material widely exploited in food and cosmetics industries and will be discussed in the last section of this review.

### 2.4. Bacterial Cellulose

Microbial polysaccharides represent promising and cheap biomaterials with potential commercial exploitation. Among them, bacterial cellulose (BC) is receiving major attention (Figure 2), thanks to its potential fields of application and low production costs [57]. In 1883, Brown was the first to report the biosynthesis of cellulose by Acetobacter xylinum¸ in aerobic conditions using glucose as carbon source [58]. Nowadays, bacteria can be easily genetically modified in order to maximize cellulose productions using biotechnology techniques and employing agro-industrial waste material as a bacterial growth medium [59]. Cellulose synthesized by microbial fermentations displays several advantages over plants celluloses such as the absence of lignin and other contaminants and no dependency on regional, season harvest time, and climatic conditions [59,60]. BC is a dietary fiber and its use in food industries was approved as a “generally recognized as safe” food (GRAS) by the FDA. BC was already exploited as an additive for the fabrication of chewy dessert (nata-de-Coco a worldwide diffused dessert obtained through bacteria fermentation in coconuts milk), as fat replacer (ice creams production), meat analogs, and as emulsion stabilizer [61,62]. The hydroxyl groups and crystalline structure combined with a wide number of hydrogen bonds offer BC good hydrophilicity and possible hydrophobic interactions due to its crystalline solid conformation [63,64]. BC and BC nanocrystals have been currently employed for the fabrication of micro/nanoemulsion, evidencing good results, even in the absence of hydrophobic groups within their structure that are usually required to interact with lipophilic molecules [65].

### 2.5. Bacterial Biosurfactants

Among microbial products, biosurfactants (also known as microbial surfactants) were investigated as candidate biomolecules for cosmetic applications [66]. Biosurfactants are surface-active materials produced by microorganisms such as yeast, bacteria, and fungi. Such mixtures are commonly composed of a large variety of chemicals such as glycolipids, lipopeptides, lipoproteins, fatty acids, polysaccharides, and phospholipids. Among them, glycolipids are the most investigated compounds since they present an amphiphilic nature provided by an apolar hydrophobic tail combined with a polar head mainly constituted by carbohydrate moieties [67]. Biosurfactants have shown promising applications as detergent, emulsifier, wetting and foaming agents and to promote the solubilization of hydrophobic substances [68].

### 2.6. Hyaluronic Acid

Hyaluronic acid (HA) (also known as hyaluronan) is a linear, negatively charged polysaccharide, constituted by repeating β-1,4-d-glucuronic acid and β-1,3-*N*-acetyl-d-glucosamine disaccharide units (Figure 2). HA is one of the most exploited polysaccharides for the fabrication of beauty care products, thanks to the high hydrophilicity and its ability to retain water. However, the use of hyaluronan to date is mainly limited to topical application as a body cream additive, contributing to the flexibility of healthy skin field [69]. Very few works report an efficient use of HA-nanostructures for the encapsulation of cosmetic agents; indeed, only recently a nanostructured polyelectrolyte complex was obtained by employing the polycation chitosan. The developed nanocomplex was tested as an encapsulating agent for model molecules (menthol and eugenol), displaying moderate loading and promising resistance against microorganism deterioration provided by chitosan activity [70].

## 3. Marine Polysaccharides

Marine organisms represent rich sources of structurally diverse biologically active compounds such as fatty acids, polysaccharides, essential minerals and vitamins, enzymes, pigments, and peptides with a promising cosmeceutical potential [71]. Unlike most natural sources exploited and discussed above, which are commonly farmed or produced, most marine natural sources exploited for food and cosmetic industries are abundant and generally considered as waste material [2,71]. Their possible exploitation to obtain high-value chemicals represents an important scientific challenge and ethical issue, particularly in the present period of strong economic, food, and worldwide environmental crises. The present paragraph reports a brief overview of the most promising polysaccharides obtained from marine organisms such as crustacean and seaweed (Figure 3).

### 3.1. Chitin and Chitosan

Chitosan is a semi-synthetic polysaccharide obtained through the deacetylation of chitin, a major component of the exoskeletons of crustaceans such as crab, shrimp, and crawfish. The polysaccharide is a copolymer of β-(1,4)-linked d-glucosamine (deacetylated unit) and *N*-acetyl-d glucosamine (acetylated unit) (Figure 3). Chitosan has been widely exploited in the biomedical field, thanks to its intrinsic antimicrobial activity toward pathogenic bacteria [72]. Chitosan antimicrobial activity made it a perfect additive for product preservation and food packaging and is currently employed as film a coating or nanostructured system [73]. Chitosan nanoparticles are typically prepared through the electrostatic interaction between amine groups of chitosan and the negatively charged groups of a polyanion such as tripolyphosphate. In 2015, Fakhreddin was the first to design an edible film based on gelatin reinforced with chitosan nanoparticles in order to avoid food spoilage caused by bacterial deterioration without compromising the taste and integrity of protected foods [74]. Besides its antimicrobial activity, the good encapsulation efficiency of chitosan nanoparticles was explored for the development of cosmetic products such as sunscreen, hair beauty, and teeth care formulations [75]; as well as for food applications, with several active agents such as annatto (typically used in food industries as natural condiment food coloring), saffron, and essential oils (Mentha piperita) entrapped into chitosan nanoparticles [76,77]. The use of chitosan-based formulations is also accepted for patients allergic to crustaceans, thanks to the growing availability of chitosan obtained from fungi [78].

### 3.2. Seaweed Polysaccharide

Macroalgae are abundant biomass and most of the naturally produced and harvested algal biomasses are left to decompose on the shore, creating several waste problems [79]. Nevertheless, algae could represent an ideal renewable resource of biomaterials [80]. Their current use is mainly limited to food consumption, but they possess potentiality as a renewable and sustainable feedstock of bioactive molecules such as fatty acid, proteins, and in particular polysaccharides whose amount could reach 70% of total dry weight [81]. Most seaweed polysaccharides, obtained from green (ulvan), brown (fucoidan and alginate), and red (carrageen) algae, play a fundamental role as structural components in plant cell wall [82]. Such natural polymers are currently classified as dietary fibers since they cannot be digested by human metabolism. However, the regular assumption of dietary fibers has been confirmed to have several beneficial effects on human health, such as the reduction of risk of colon cancer, hypercholesterolemia, and diabetes [83]. Alginate, obtained from brown algae such as Laminaria spp, Ascophyllum nodosum, and Ecklonia maxima, is an anionic copolymer of β-1,4-d-mannuronic acid and α-l-guluronic acid (Figure 3). Its exploitation in food industries is well-known, even in molecular gastronomy, where alginate macroscopical beads are produced without the necessity of laboratory equipment by dropping a water alginate suspension into a divalent cations solution (usually CaCl_2_) [84]. However, the gelation technique allows development of structures even on a micro/nanoscale through the optimization of reaction conditions. Active agents can be easily loaded into alginate nanoparticles through their addition in the reaction mixture [85].

#### Sulphated Polysaccharide

Among seaweed polysaccharides, sulphated polysaccharides have emerged as a promising biomaterial for the preparation of edible and biocompatible micro/nanostructures [86]. The use of sulphated polysaccharides as an emulsifier is still poorly investigated due to the high hydrophilicity that hamper their interactions with hydrophobic molecules [52]. However, the high content of bonded proteins within their chemical structures and the presence of few hydrophobic groups can promote the formation of stable oil in water emulsions.

Carrageenan extracted from red algae is employed as an emulsion stabilizer in virtue of its gelling and thickening properties [87]. Notwithstanding its widely reported bioactivity, recent studies have shown the induction of adverse effects in living organisms in response to the systemic oral administration of carrageenan formulation [88]. However, to date, carrageenan included in cosmetic products was not reported to induce adverse effects on human skin [89].

Fucoidans obtained from brown seaweeds show stronger emulsifying properties, compared to carrageenan, due to their protein content and mild amphiphilic character provided by the simultaneous presence of hydrophobic groups (methyl) and hydrophilic groups within their polymeric backbone [90,91]. Moreover, a recent study demonstrated the strong antioxidant activity of fucoidan [92]. Such antioxidant properties seem to be strongly correlated to the molecular weight, degree of sulfation, and position of the sulphated groups [91]. Furthermore, stronger antioxidant activities were observed in correlation with a higher content of polyphenol and protein in crude extracts [93]. Such features open new opportunities regarding its application for the preservation of bioactive molecules susceptible to degradation caused by oxidizing agents.

Finally, ulvan, a sulphated heteropolysaccharide extracted from edible green algae belonging to Ulva spp, thanks to its presence in the polymeric structure of hydrophilic (hydroxyl, sulfate and carboxyl) and hydrophic (methyl) groups, displays an amphiphilic character exploited as an emulsifier for the development of emulsions for food and cosmetic applications [52,94]. The surfactant activity of ulvan as an emulsion stabilizer could be further enhanced by the high protein fraction, which is reported to be indissolubly bound to the polysaccharide structure even after the extraction process [79]. In addition, ulvan polysaccharide displays antioxidant activity similar to fucoidans, correlated to sulphate content [95]. Concerning worldwide availability, sulphated polysaccharides obtained from algae biomass represent one of the most important classes of renewable polymers with promising potential in several research fields.

## 4. Modified Polysaccharides

Polysaccharides, thanks to their presence in the repeating units of reactive groups such as amino, carboxyl and/or hydroxyl, are often submitted to chemical modification to improve their performance in the development of novel nano/microsystems for the food and cosmetic industries.

The hydrophilic/hydrophobic balance of polysaccharides can be properly balanced by improving the hydrophobic counterpart of the macromolecule by introducing apolar groups. Starches are commonly weak emulsifiers due to their high hydrophilicity provided by the glucose backbone; however their chemical modification achieved by introducing hydrophobic components represent a common and diffused procedures to enhance the amphiphilic character [96]. Indeed, starch sodium octenyl succinate (E1450) is one of the most exploited food additive in particularly in beverage productions, where is employed as emulsifier in virtue of its enhanced amphiphilic profile provided by the addition of hydrophobic groups [97]. The introduction of apolar chains is generally achieved through the esterification reaction, exploiting the hydroxyl groups of the polysaccharides [98]. In this respect, several polysaccharides have already been submitted to chemical modifications; for example, various derivate cellulose ethers (methylcellulose, carboxymethyl cellulose, and hydroxypropyl cellulose) have found several applications in food (ice cream production), cosmetic (shampoos and toothpaste stabilizer), and medical (protective colloidal agents) industries [98]. Cationic polysaccharides, such as chitosan, represent an attractive class of polymers in virtue of their high affinity to anionic substrates such as skin and hair, thus being very effective as conditioning cosmetic agents [99]. Moreover, the presence of charged groups allows to develop self-assembling micro/nanostructures by electrostatic interaction with opposite charge macromolecules, leading to the formation of polyelectrolyte complexes (Figure 1). Cationization reactions have become an effective modification procedure to enhance the potential applications of various polysaccharides. The most cationized polysaccharides are starches whose procedure of modification generally involves the use of 3-chloro-2-hydroxypropyltrimethylammonium chloride (CHTAC) or 2,3-epoxypropyltrimethylammonium chloride (ETAC); an increasing number of papers and patents have been already published reporting their potential uses in the food and cosmetic industries [100]. Although chemical modification methods are commonly employed, the presence of organic and toxic reagents may discourage their application in particular in food science, where mild and un-expensive procedures, combined with the absence of possible toxic contaminants, are commonly required. In this respect, enzymatic bioprocesses have been investigated as potential alternatives to chemical approaches in virtue of their high specificity, production yield, and mild reaction conditions. Notwithstanding their potential applications, the cost of most of the enzymatic reactions are still too high to be industrially sustainable and their use for polysaccharide modification is still not well investigated. To date, only few examples report the use of lipase to improve the hydrophobicity of polysaccharides to be applied as a novel emulsifier [101]. One of the most common lipase products is starch sodium octenyl succinate, whose production can be achieved by lipase-coupling esterification in aqueous environments [102]. The above described strategies display potential advantages to modify polysaccharides features, providing novel properties or enhancing those already owned; however, it is important to emphasize that all procedures must respect the strict normative of safety in order to avoid any potential risk to the consumer.

## 5. Conclusions

The exploitation of natural polymers, especially renewable polysaccharides, as a natural alternative to conventional synthetic-based materials for micro/nanostructures development and/or as emulsifying agents, could represent a promising breakthrough for food and cosmetic applications. The industrially feasible manufacturing of food and cosmetics products based on such polymers would contribute to addressing the issue related to the increasing consumer demand for novel free-label formulas. An essential advantage of renewable polysaccharides is their sustainability, since they can be obtained from abundant, and often detrimental, waste biomass. However, a major drawback common to natural polymers is the lack of reproducibility of the materials’ chemical composition upon extraction. Thus, efforts should be devoted to standardizing easy and environmentally sustainable isolation and modification procedures aimed at minimizing the differences of chemical compositions of the extracted biopolymers. This will significantly contribute to triggering their industrial feasibility as a basic component of micro/nanoformulations for food and cosmetic applications.
